# Identification of Potential Biomarkers for Patients with DWI-Negative Ischemic Stroke

**DOI:** 10.1007/s12031-024-02229-z

**Published:** 2024-07-12

**Authors:** Lei Li, Ying Wang

**Affiliations:** Department of Neurology, The Second Affiliated Hospital of Kunming Medical University, Kunming, 6500032 China

**Keywords:** Ischemic stroke, Diffusion weighted imaging, Gene pair, Immune biomarkers, Metabolism biomarkers

## Abstract

Ischemic stroke is the leading cause of long-term disability in adults, accounting for 80% of stroke cases. Diffusion weighted imaging (DWI) examination is the main test for acute ischemic stroke, but in recent years, several studies have shown that some patients show negative DWI examination after the onset of ischemic stroke with symptoms of significant neurological deficits. In this study, we investigated potential biomarkers related to immune metabolism in the peripheral blood of DWI-negative versus DWI-positive patients after ischemic stroke and explored their possible regulatory processes in ischemic stroke. The datasets related to ischemic stroke were downloaded from the GEO database, immune-related genes and metabolism-related genes were obtained from the ImmPort database and MSigDB database, respectively, and immune-related differential genes were obtained based on immune scores using the algorithm of the R software package “GSVA.” Candidate genes were selected based on intersections, hub genes were screened using the algorithm in Cytoscape software, and finally, GeneMANIA analysis, GSEA enrichment analysis, subcellular localization, gene transcription factor and gene-drug interaction networks, and disease correlation analyses were performed for the hub genes. Five hub genes (GART, TYMS, PPAT, CTPS1, and PAICS) were obtained by PPI network analysis and software analysis. Among them, PPAT and PAICS may be the real hub genes with consistent and significantly differentiated results from the discovery and validation sets. The functions of these hub genes may be related to pathways such as nucleotide biosynthetic processes. The constructed hub gene ceRNA network showed that hsa-10a-5p is the key miRNA connecting PAICS and multiple lncRNAs in this study. Differential genes related to immunity and metabolism in DWI-negative and DWI-positive patients after IS were identified using bioinformatics analysis, and their pathways and related TF-RNAs, miRNAs, and lncRNAs were identified. These genes may be considered effective targets for the diagnosis and treatment of ischemic stroke.

**Supplementary Information:**

The online version contains supplementary material available at 10.1007/s12031-024-02229-z.

Ischemic stroke (IS) is the most common disease in neurology. It is the second-leading cause of death worldwide and the third-leading cause of death and disability combined (Collaborators et al. 2021). IS is characterized by a high incidence rate, disability rate, mortality rate, and recurrence rate (Donkor 2018). The global estimated lifetime risk of stroke increased from 22.8% in 1990 to 24.9% in 2016, and the highest risk in China was estimated as 39.3% (Feigin et al. 2018). This may be closely related to the incidence rate of hypertension, diabetes, hyperlipidemia, hyperhomocysteinemia, and bad life habits, such as staying up late and consuming a high-fat diet.

Diffusion weighted imaging (DWI) is currently the only noninvasive method that can detect water and diffusion movement in living tissues. It has unique imaging features in acute ischemic stroke (AIS) and is of great significance for the early diagnosis of AIS. However, multiple studies have shown that not all AIS patients are DWI positive. A study in 2017 involving 3236 patients with clinically suspected AIS and complete DWI examination within 72 h of onset showed that approximately 6.8% of patients had negative DWI, and DWI-positive patients’ symptoms of posterior circulation infarction were five times higher than those of anterior circulation (Edlow et al. 2017). The characteristics of DWI-negative patients can be summarized as follows: (1) minor stroke (NIHSS score ≤ 3), (2) hyperacute ischemia within 6 h of onset, and (3) TOAST classification: large atherosclerosis stenosis/occlusion or arteriole occlusion (Edlow et al. 2017; Oppenheim et al. 2000). In recent years, researchers have begun to explore other biological or serological indicators of DWI-negative patients. Plasma osmotic pressure and homocysteine levels are considered possible specific indicators of DWI-negative patients (Aiba et al. 2019; Yuan et al. 2019). In summary, seeking new biological or serological markers for DWI-negative AIS patients is crucial for the diagnosis of IS.

IS refers to neurological dysfunction caused by the interruption of cerebral arterial blood flow for various reasons. Its pathophysiological mechanism mainly includes reactive oxygen species generation, inflammatory cell infiltration, breakdown of the blood‒brain barrier (BBB), and irreversible necrosis of neurons. Research shows that immune cells (including neutrophils, T cells, B cells, dendritic cells, and macrophages) after cerebral ischemia are rapidly activated and fully participate in the acute and chronic phases of ischemic cerebral apoplexy. Immune cells are not only significantly related to long-term problems, such as depression and dementia (Iadecola et al. 2020), but also reduce or exacerbate brain damage by affecting the expression of inflammatory factors and the release of cytotoxic factors (Jian et al. 2019). Recent studies have shown that IS can lead to a reduction in spleen volume and a sharp reduction in splenocytes, but the number of CD4 + FoxP3 + Treg cells and circulating CD11b + macrophages/dendritic cells (DCs) increases. This can lead to severe immunosuppression and may trigger potential systemic infections (Prass et al. 2003; Ross et al. 2007). After the occurrence of cerebral ischemia, brain tissue cells affected by ischemia are damaged by hypoxia, resulting in a series of stress reactions. As nerve cells die in the central area of the infarction, a large number of microglia and macrophages of the central nervous system are activated, producing a series of markers and morphological changes, including CD16, CD32, CD86, and integrin α-M. Macrophage mannose receptor 1 (CD206) is involved in tissue damage (proinflammatory M1 phenotype) and repair (anti-inflammatory M2 phenotype) (Jiang et al. 2020). Additionally, after IS metabolic biomarkers such as cysteine, glutamine, and adenosine reduced and aggravated brain damage by affecting mitochondrial and DNA damage (Shin et al. 2020). Therefore, in addition to conventional treatment, immunomodulatory and metabolic therapy as alternative treatment methods is worthy of in-depth research.

Our research aims to identify differentially expressed genes between DWI-negative and DWI-positive (control) patients after IS, with a focus on immune-related genes (IRGs) and metabolism-related genes (MRGs), and further explore the potential mechanisms of these new biomarkers from the perspective of cellular immunity and cellular metabolism. Thus, this study aims to provide better guidance for the diagnosis of AIS and support the search for potential targets for IS treatment.

## Methods

### Sequencing Analysis

In this study, whole blood specimens were collected from five patients with negative DWI examination after IS and four patients with positive DWI examination after IS (DWI was completed within 3 days of onset in all patients). The patients were as follows: (1) negative DWI specimens after IS: DWI NEG1, DWI NEG2, DWI NEG3, DWI NEG4, and DWI NEG5; and (2) positive DWI specimens after IS: DWI POS1, DWI POS2, DWI POS3, and DWI POS4. Whole transcriptome sequencing data of the samples were collected through the clinic. To mitigate the sequencing errors of the samples due to the combined effects of multiple factors, such as the sequencer itself, sequencing reagents, and the samples, and to obtain high-quality reads, we utilized HISAT2 to sequence the clean reads against the formulated reference genomes by using HISAT2 (vision: 2.1.0) (http://www.ccb.jhu.edu/software/hisat/) (Kim et al. 2015). The reference genomes or the gene position information and sequence feature information specific to the sequenced samples were obtained for quality control of the samples. The reads that were strictly filtered and detected were called clean reads, and all the subsequent analyzed results were based on the clean reads (Supplementary Fig. 1). Using the known reference gene sequences and annotation files as a database, sequence similarity comparison was adopted to identify the expression abundance of each protein-coding gene in each sample. HTSeq-Counts software (version: 2.0.4) (https://pypi.python.org/pypi/HTSeq) (Anders et al. 2015) was used to obtain the number of reads on the compared to protein coding genes in each sample. Then, the FPKM value of the expression of the protein coding genes was calculated based on the FPKM consensus.

**Fig. 1:**
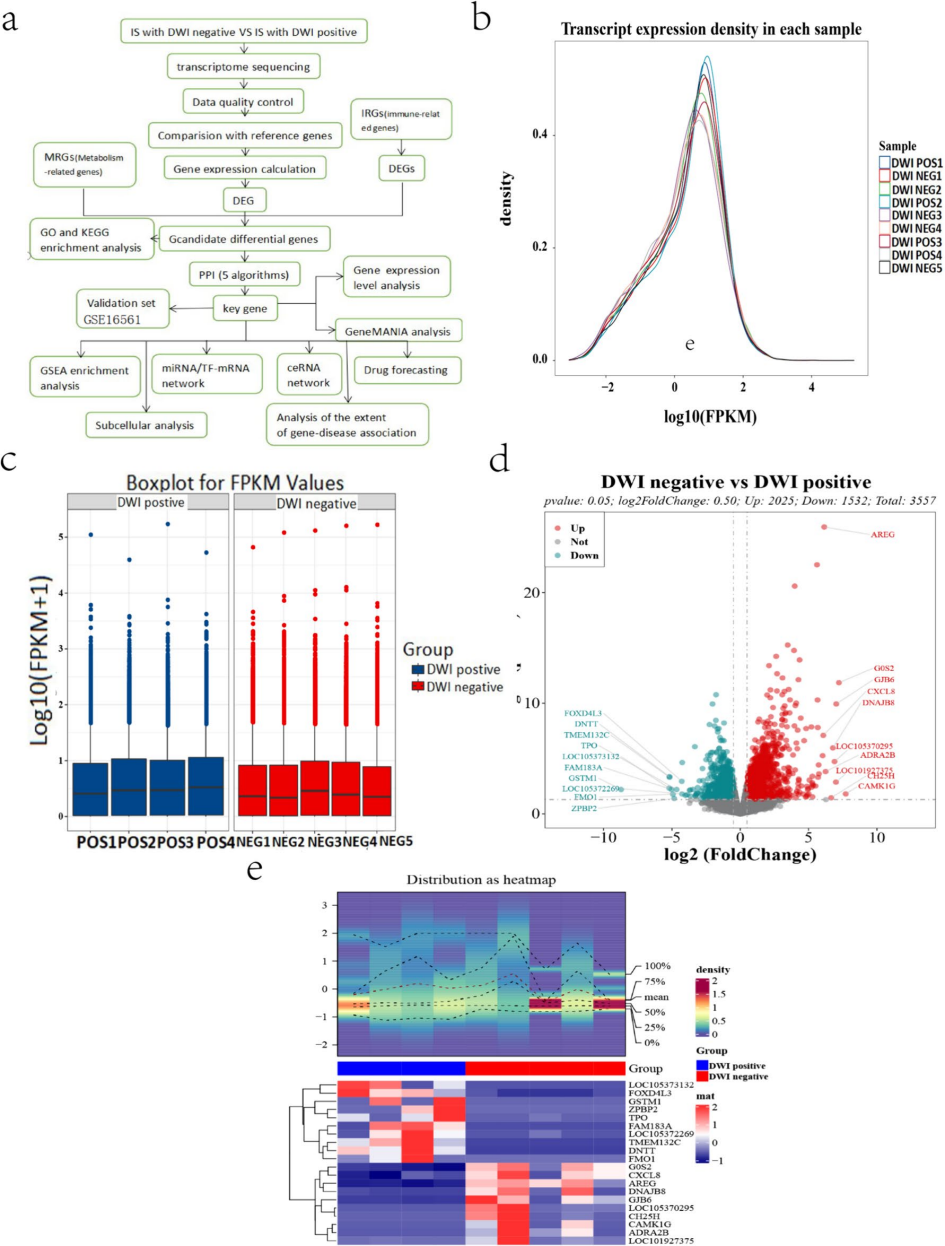
**a** Technical roadmap. **b** Overall distribution of sample gene expression energy. **c** Box line plot of sample expression. **d** Volcano plot based on the distribution of differentially expressed genes between DWI-negative and DWI-positive specimens (red dots are upregulated genes, green dots are downregulated genes, and gray dots are not differentially expressed genes). **e** Circle plot based on differentially expressed genes between DWI-negative and DWI-positive patients

Clinically collected DWI-negative and DWI-positive specimens after IS were used as a discovery set. Because no dataset has been found to correlate with DWI examinations, which are all based on patients with IS, we downloaded GSE16561 microarray expression data (peripheral whole blood) from the GEO database (https://www.ncbi.nlm.nih.gov/geo/) (Supplementary Table 1). The peripheral blood samples from 39 IS patients and 24 healthy control individuals (control) were selected for subsequent validation. A total of 1039 immune-related genes (IRGs) were obtained by downloading immune-related genes from ImmPort data (https://www.immport.org/home) (Supplementary Table 2); a search from the Molecular Signatures Database (MSigDB, https://www.gsea-msigdb.org/) with the keyword “metabolism” yielded approximately 948 metabolism-related genes (MRGs) (Supplementary Table 3).

**Table 1:** Differentially expressed genes of DESeq2 samples

Number	Upregulated gene	Downregulated gene
1	CAMK1G	GSTM1
2	G0S2	LOC105373132
3	GJB6	DNTT
4	LOC101927375	TMEM132C
5	LOC105370295	FAM183A
6	DNAJB8	FMO1
7	CH25H	ZPBP2
8	ADRA2B	TPO
9	AREG	FOXD4L3
10	CXCL8	LOC105372269

**Table 2:** Sample immune and metabolic genes differentially expressed genes

Number	Upregulated gene	Downregulated gene
1	CAMK1G	GSTM1
2	CH25H	LOC105373132
3	ARSI	DNTT
4	ADRA2B	TMEM132C
5	LOC101927375	FAM183A
6	LOC105370295	CLDN10
7	DNAJB8	FMO1
8	NR4A3	GTSF1L
9	POU2F3	OIT3
10	PNCK	NEUROD4

**Table 3:** Candidate gene GO and KEGG enrichment analysis

Number	Biological process (BP)	Cellular component (CC)	Molecular function (MF)	KEGG
1	Cellular amino acid metabolic process	Mitochondrial matrix	Phosphoric ester hydrolase activity	Purine metabolism
2	Ribonucleotide metabolic process	Mitochondrial inner membrane	Acyltransferase activity	Amino sugar and nucleotide sugar metabolism
3	Ribose phosphate metabolic process	Organelle outer membrane	Acyltransferase activity, transferring groups other than amino-acyl	Biosynthesis of cofactors
4	Alpha-amino acid metabolic process	Outer membrane	Lyase activity	Nucleotide metabolism
5	Purine nucleotide metabolic process	Peroxisomal matrix	Phosphoric diester hydrolase activity	Pyrimidine metabolism
6	Small molecule catabolic process	Microbody lumen	Acetyltransferase activity	Glycine, serine, and threonine metabolism
7	Organophosphate catabolic process		Monooxygenase activity	Tryptophan metabolism
8	Carboxylic acid catabolic process		3,5-Cyclic-AMP phosphodiesterase activity	Chemical carcinogenesis-DNA adducts
9	Amino sugar biosynthetic process		3,5-Cyclic-nucleotide phosphodiesterase activity	Drug metabolism-cytochrome P450
10	UDP-N-acetylglucosamine metabolic process		Cyclic-nucleotide phosphodiesterase activity	Biosynthesis of amino acids

### Differential Expression Analysis

Differential expression analysis was performed between DWI-negative and DWI-positive patients based on the sample expression matrix (HTSeq-Counts) using the R package “DESeq2” (version: 1.40.2) (http://www.bioconductor.org/packages/release/bioc/html/DESeq2.html) (Love et al. 2014), using *p* value < 0.05 and |log2FoldChange|> 0.5 as the thresholds to screen for differentially expressed genes.

### Immune-Related and Metabolism-Related Differential Gene Identification to Obtain Candidate Genes

The single sample gene set enrichment analysis (ssGSEA) algorithm of the R package “GSVA” (version: 1.28.0) (http://www.bioconductor.org/packages/release/bioc/html/EGSEA.html) (Hanzelmann et al. 2013) was used to calculate the immune-related scores of all samples based on 1039 immune-related genes (IRGs), and the ssGSEA scores of all samples were calculated using the R package “GSVA.” The ssGSEA score was calculated based on 1039 IRGs. The samples were categorized into high- and low-scoring groups based on the median ssGSEA scores of all samples. Candidate genes were identified by intersection using the R package “VennDiagram” (version: 1.7.1) (https://CRAN.R-project.org/package=VennDiagram) (Chen and Boutros 2011).

### Candidate Gene Functional Enrichment Analysis

Candidate genes were analyzed by GO and KEGG enrichment using the R package “clusterProfiler” (version: 4.0) (https://github.com/GuangchuangYu/enrichment4GTEx_clusterProfiler) (Wu et al. 2021). GO (Gene Ontology) enrichment analysis is a method to functionally annotate genes according to the GO database, which includes three parts: biological processes (BPs), cellular components (CCs), and molecular functions (MFs). KEGG pathway analysis is a method to annotate the pathways of all identified proteins or screened differentially expressed proteins with the help of the KEGG database and analyze the most important metabolic and signaling pathways in which these proteins or genes are involved.

### PPI Interaction Network Screening for Hub Genes

Based on the candidate genes, PPI network analysis was performed using protein interaction information from the STRING database (https://string-db.org/) (set medium confidence > 0.4 to remove isolated targets). The protein interaction information was imported into Cytoscape software to establish the candidate gene protein interaction network. The cytoHubba plug-in in Cytoscape software was utilized to build the candidate protein interaction network based on nine algorithms: MCC (maximal clique centrality), MNC (maximum neighborhood component), degree, EPC (edge percolated component), EcCentricity, CTC, and EPC (edge percolated component). EcCentricity, Closeness, Radiality, Betweenness, and Stree were used to calculate gene nodes and further filter hub genes.

### Hub Gene and Enrichment Analysis

Coexpression networks and related functions of hub genes were analyzed based on the GeneMANIA database (http://genemania.org/). The samples were categorized into high and low-expression groups of the hub genes. The R package “DESeq2” was used to analyze the differences between the high and low-expression groups in the discovery set (high- vs. low-expression groups), and the logFC was calculated. The logFCs were sorted from the largest to the smallest and then enriched by GSEA. The reference gene set was the C2:KEGG gene sets in the MSigDB database (www.gsea-msigdb.org/gsea/msigdb/). Signaling pathways significantly enriched for hub genes were explored using *p*.adjust < 0.05.

### Analysis of Hub Gene Subcellular Localization, Gene Potential Drug Prediction, and Disease Relevance

The FASTA sequences of the hub genes were obtained from the NCBI website (https://www.ncbi.nlm.nih.gov/). The FASTA sequences of the hub genes were then entered into the mRNALocater database to obtain predicted scores for the five subcellular localizations of each hub gene, with the highest score being used for the final specific localization.

Prediction of small molecule drugs corresponding to hub genes was performed using the DGidb database (https://dgidb.org/). The analysis of the extent of hub gene-disease associations was performed using the CTD database (https://ctdbase.org/); CTD is a publicly available database that examines associations based on chemicals, genes, phenotypes, diseases, and environments. It can contribute to the understanding of chemical drugs and human health. The CTD database was used to predict IS and inflammation-related diseases corresponding to five hub genes (GART, TYMS, PPAT, CTPS1, and PAICS).

### TF-mRNA, miRNA‒mRNA, and ceRNA Network Construction and Expression Verification

The transcription factor (TF) corresponding to the hub gene was predicted using the NetworkAnalyst platform (https://www.networkanalyst.ca/) and the JASPAR database; the TarBase miRTarBase database was selected using the NetworkAnalyst platform to predict the hub genes. The miRNAs corresponding to hub genes were predicted using miRNet (https://www.mirnet.ca/) and the miRcode database (miRcode); based on the miRNAs shared by miRNA-mRNAs and miRNA-lncRNAs, we constructed the complete lncRNA‒miRNA-mRNA regulatory network.

Finally, the differential profile of the hub genes in DWI-negative and DWI-positive patients in the validation set (GSE16561) was explored. The differential profile of hub genes in the discovery and validation sets was constructed to draw conclusions. The technology roadmap is shown in Fig. 1a.

## Results

### Differential Expression Analysis

We first estimated the expression levels of protein-coding genes by counting sequenced reads localized to the genomic regions or exonic regions of protein-coding genes. The HTSeq-Count software was used to obtain the number of reads paired to the protein-coding genes in the nine samples, and Cufflinks software (version: 2.2.1) (http://cole-trapnell-lab.github.io/cufflinks/) (Trapnell et al. 2012) was used to calculate the FPKM values of protein-coding gene expression (Supplementary Table 4). The overall distribution of the sample genes is shown in Fig. 1b, and the expression of the sample genes was basically at a uniform level after normality treatment (Fig. 1c), which is comparable. Differentially expressed genes were identified in the above 9 samples. Differentially expressed genes were screened with *p* value < 0.05 and log2FoldChange > 0.5 as the threshold. The sample expression matrix (HTSeq-Counts) of the R package “DESeq2” yielded 3557 differentially expressed genes (Supplementary Table 5 and Supplementary Table 6), including 2025 upregulated genes and 1532 downregulated genes (Fig. 1d). The differential folds according to log2(FoldChange) are shown in the volcano plot. The expression relationships were verified by their expression densities for the first ten genes (Table 1), which are shown in a heatmap (version: 2.17.0) (http://www.bioconductor.org/packages/devel/bioc/html/ComplexHeatmap.html) (Gu et al. 2016) (Fig. 1e).

**Table 4:** Predicted scores of hub gene subcellular localization

Gene	mRNALocater	Cytoplasm	Endoplasmic reticulum	Extracellular region	Mitochondria	Nucleus
GART	Nucleus	0.2473	0.1302	0.0301	0.013	0.5794
TYMS	Nucleus	0.3625	0.1138	0.0728	0.02	0.4309
PPAT	Nucleus	0.2841	0.2578	0.0261	0.0133	0.4186
CTPSI	Nucleus	0.118	0.0508	0.0409	0.0107	0.7796
PAICS	Cytoplasm	0.5936	0.2159	0.0234	0.0105	0.1565

**Table 5:** Correspondence between hub genes and drugs

Gene	Drug	Sources
GERT	Pemetrexed	TdgClinicalTrial|TEND
GERT	Peltrexol	TTD
GERT	Lometrexol	DTC
TYMS	OSI-7904	TdgClinicalTrial
TYMS	Capecitabine	ClearityFoundationBiomarkers|ClearityFoundationClinicalTrial|ChemblInteractions
TYMS	Methotrexate	CIViC
TYMS	Irinotecan	CIViC|PharmGKB
TYMS	Hydrocortisone	NCI
TYMS	Tegafur	TdgClinicalTrial
TYMS	Fluorouracil	DTC|ClearityFoundationClinicalTrial|ChemblInteractions|CIViC
TYMS	Pemetrexed	ClearityFoundationBiomarkers|TdgClinicalTrial|TEND|CIViC
TYMS	Raltitrexed	TdgClinicalTrial|TEND|PharmGKB
TYMS	Indomethacin	NCI
TYMS	Tamoxifen	NCI
TYMS	Dexamethasone	NCI|PharmGKB
TYMS	Hydroxychloroquine	PharmGKB
TYMS	Asparaginase	PharmGKB
TYMS	Daunorubicin	NCI|PharmGKB
TYMS	Cisplatin	NCI
TYMS	Deoxyuridine monophosphate	DTC
TYMS	Azathioprine	PharmGKB
TYMS	Floxuridine	TdgClinicalTrial|ClearityFoundationClinicalTrial|ChemblInteractions|TEND
TYMS	Arfolitixorin	TdgClinicalTrial
TYMS	9-Aminocamptothecin	NCI
TYMS	Platinum	PharmGKB
TYMS	Leucovorin	TdgClinicalTrial|TEND
TYMS	Doxifluridine	NCI
TYMS	Reserpine	NCI
TYMS	Topotecan	NCI
TYMS	Prednisone	PharmGKB
TYMS	Azacitidine	PharmGKB
TYMS	Eniluracil	NCI
TYMS	Carbogen	NCI
TYMS	Phentolamine	NCI
TYMS	Sulfasalazine	PharmGKB
TYMS	Etoposide	PharmGKB
TYMS	Cytarabine	PharmGKB
TYMS	Vincristine	NCI
TYMS	Thymidine monophosphate	DTC
TYMS	Verapamil	NCI
PPAT	Azathioprine	ChemblInteractions|TTD
PPAT	Azathioprine sodium	ChemblInteractions
PPAT	Mercaptopurine	TTD
PPAT	Mercaptopurine	ChemblInteractions

**Table 6:** Hub genes and predicted TFRNA

Hub genes	Predicted TFRNA
CTPS1	FOXA1, GATA2, PAX2
GART	E2F1, FOXC1, FOXL1, GATA2, HINFP, NFIC, PRRX2, RELA, SRF
PAICS	BRCA1, CREB1, E2F4, GATA1, GATA2, MAX, MEF2A, NR2F1, NRF1, SOX17, SREBF2, TFAP2C, USF1, USF2, YY1
PPAT	CREB1, E2F1, GAATA2, GATA3, MAX, NRF1, RELA, SREBF1, USF1, USF2
TYMS	BRCA1, EN1, ESR1, FOXC1, GATA2, GATA3, HNF4A, KLF5, NFFKB1, NR3C1, PPARG, RELA, SREBF1, TP53

### Identification of Immune- and Metabolism-Related Differentially Expressed Genes and Access to Candidate Gene

**Fig. 2:**
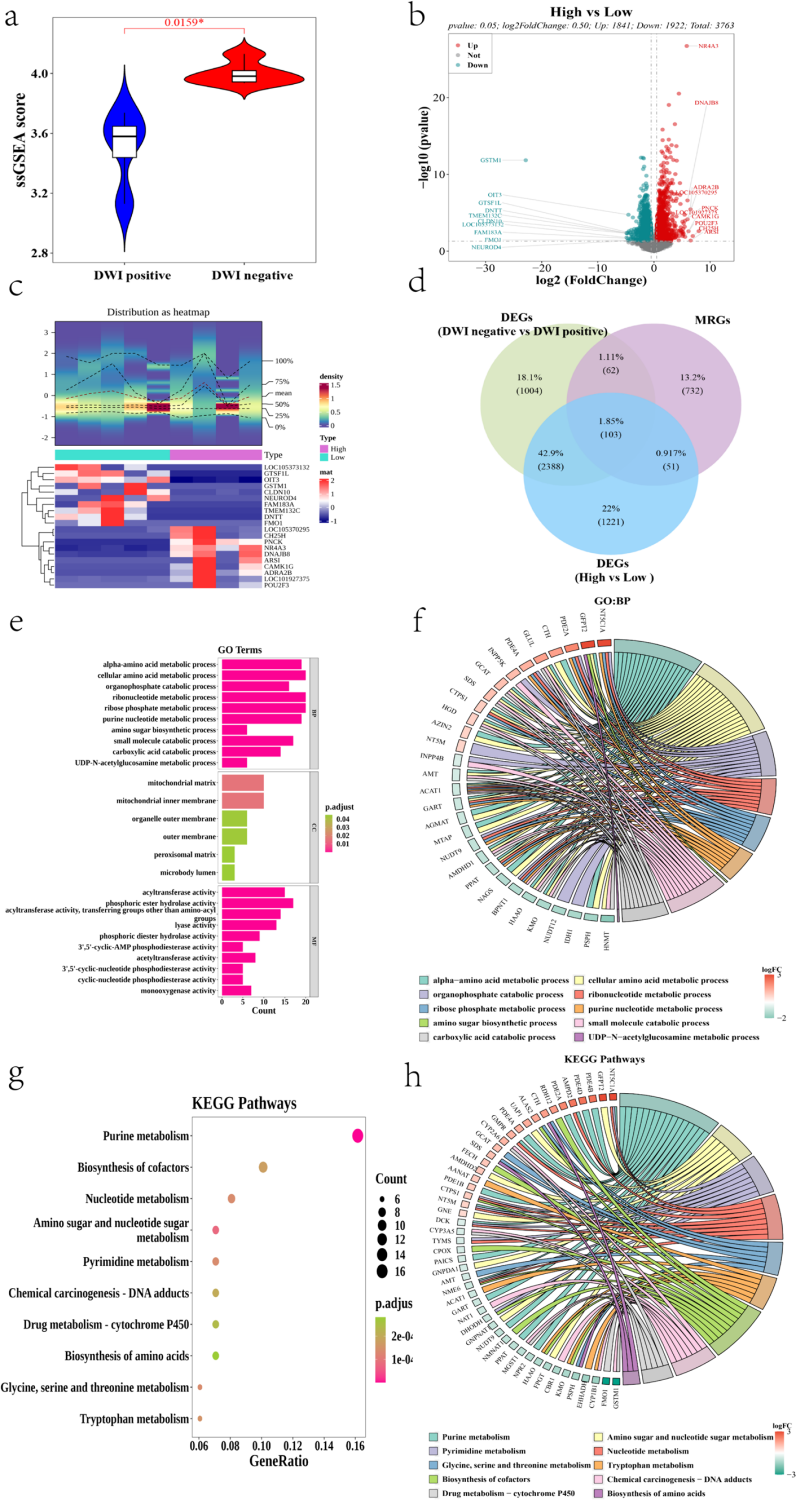
**a** Immune-related ssGSEA (single-sample gene set enrichment analysis) score line box plot between DWI-negative patients and DWI-positive samples. **b** Volcano distribution of differentially expressed genes based on high- and low-score groups (red points are upregulated genes, green points are downregulated genes, and gray points are not differentially expressed genes). **c** Circle plot of differentially expressed genes based on high- and low-score groups. **d** Intersecting Wayne plot of candidate genes. Green represents DWI negative-related differential genes, blue represents immune-related differential genes, and purple represents metabolism-related genes. **e** Candidate gene GO enrichment histogram. Horizontal coordinate is the number of genes involved in GO function, and vertical coordinate is the GO function name. The color change represents the significant *p*.adjust change: the redder the GO the more significant; the greener the GO the less significant. **f** Chord diagram of candidate gene GO:BP enrichment results. Different colors on the right represent different functions, and the color of the gene ribbon on the left represents the logFC of the gene. **g** Bubble diagram of candidate gene KEGG enrichment. Horizontal coordinate is the proportion of genes involved in the KEGG signaling pathway; vertical coordinate is the KEGG signaling pathway name. The color change represents the significant *p*.adjust change. The redder the KEGG the more significant; the greener the KEGG the less significant. The bubble size represents the number of genes involved in the signaling pathway. **h** Chord diagram of candidate gene signaling pathway enrichment results

Immune-related ssGSEA scores were calculated for all samples using the ssGSEA algorithm of the R package “GSVA” based on 1039 immune-related genes (IRGs), and significant differences (*p* < 0.05) were found between DWI-negative and DWI-positive samples (Fig. 2a). We can assume that immune-associated genes are associated with DWI negativity. The samples were divided into high- and low-scoring groups according to the median immune-related ssGSEA scores of all samples (Supplementary Table 7). Then, differential expression analysis was performed based on differential expression between high and low ssGSEA scoring samples using the sample expression matrix (HTSeq-Counts) using the R package “DESeq2,” with *p* value < 0.05 and |log2FoldChange|> 0.5 as thresholds for screening differentially expressed genes (Supplementary Table 8). A total of 3763 differentially expressed genes were identified. These included 1841 upregulated genes and 1922 downregulated genes (Fig. 2b and Supplementary Table 9). The top 10 upregulated genes and top 10 downregulated genes were sorted for validation and plotted as differentially expressed gene circles (Table 2 and Fig. 2c for details). A total of 948 metabolism-related genes (MRGs) [PMID:36923791] were obtained from the Molecular Signature Database (MSigDB, https://www.gsea-msigdb.org/) by searching the keyword “metabolism” and using the DWI-negative and positive group differential genes and high and low-scoring group differential genes. A total of 103 immune-related and metabolism-related differential genes were obtained by using the intersection between DWI-negative and DWI-positive group differential genes, high- and low-ssGSEA scoring group differential genes, and metabolism-related genes (Fig. 2d).

### Functional Enrichment Analysis of Candidate Genes

To explore the biological functions and signaling pathways related to the candidate genes, we performed GO and KEGG enrichment analyses of 103 candidate genes using the R package “clusterProfiler,” and a total of 375 GO biological functions were enriched (270 GO:BPs, 6 GO:CCs, and 99 GO:MFs) for the candidate genes (Supplementary Table 10). The top 10 most significant biological processes (GO:BPs) were plotted as a chord diagrams using the “Goplot” software (version: 1.0.2) (https://wencke.github.io/) (Walter et al. 2015) (Fig. 2f). Enrichment analysis was performed for each fraction (Fig. 2e and Table 3). The screening criteria were developed based on the *p*.adiust < 0.05, and candidate genes were enriched in a total of 32 KEGG signaling pathways (Supplementary Table 11). The top 10 most significant signaling pathways are displayed in Fig. 2 g, h and Table 3.

### PPI Interoperability Network Screening for Hub Genes

**Fig. 3:**
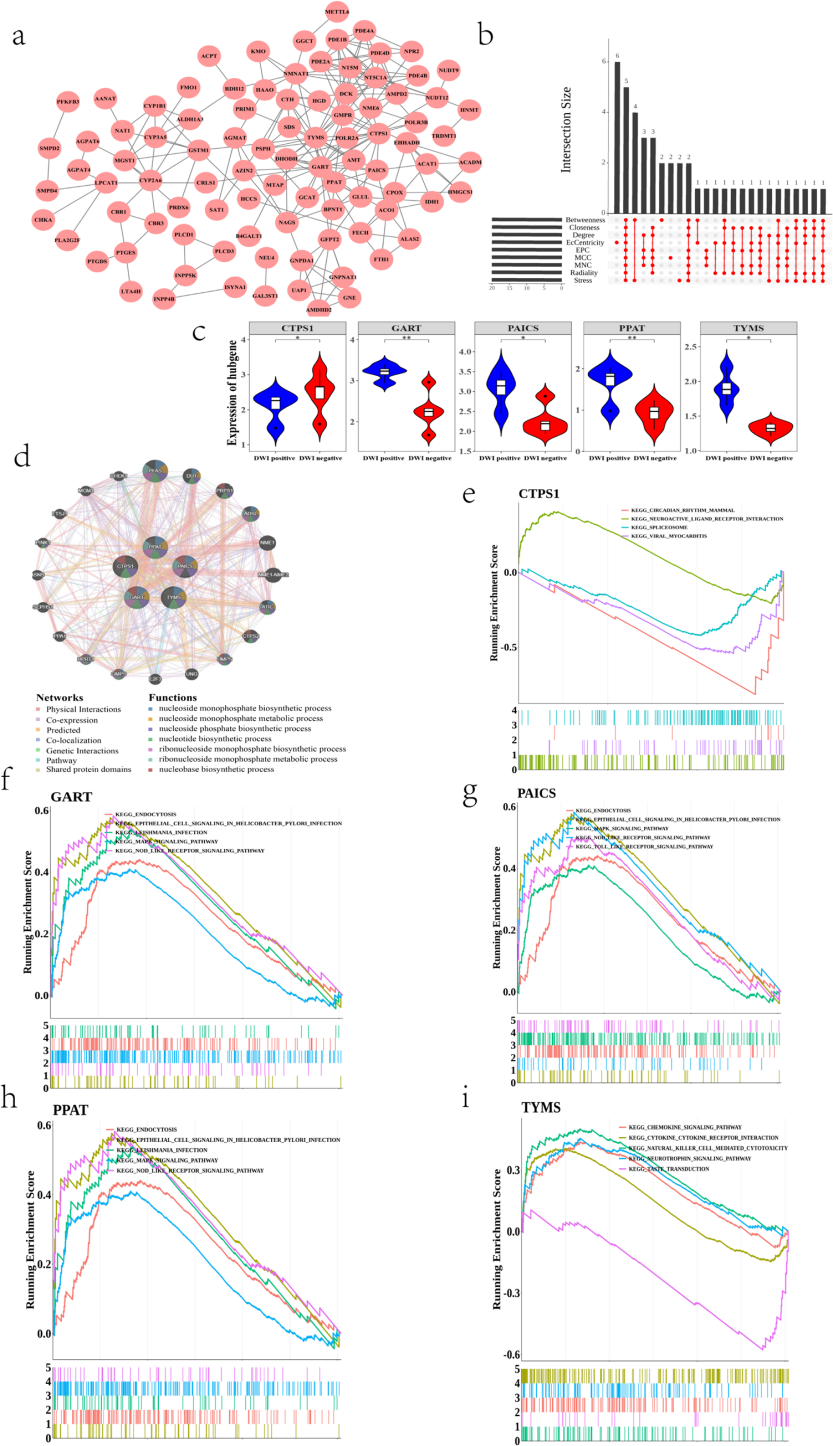
**a** PPI network map. **b** UpSet map of the top 20 genes screened using nine algorithms. **c** Box line plot of hub gene expression in the discovery set. **d** Analysis of hub genes and their coexpressed genes by GeneMANIA (center circle is five hub genes, outer circle is coexpressed genes with hub genes). **e**–**i** Hub gene enrichment signaling pathway. The top five lines are the enrichment score of the genes. The vertical axis is the corresponding running ES, and the peak in the line graph is the enrichment score of this gene set. The genes before the peak are the core genes of the gene set. The horizontal axis represents each gene in this gene set and corresponds to the vertical barcode-like lines below. The barcode-like part below is the hits, where each vertical line corresponds to a gene in this gene set

The PPI network for 103 candidate genes identified 94 nodes and 222 edges (Fig. 3a and Supplementary Table 12). Nine algorithms (MCC (maximal clique centrality), MNC (maximum neighborhood component), Degree, EPC (edge percolated component), EcCentricity, Closeness, Radiality, Betweenness, and Stree) were employed using Cytoscape software and the cytoHubba plug-in to calculate gene node scores, and the top 20 genes were selected for each algorithm. The top 20 genes with node scores for each algorithm were screened and plotted in the UpSet graph (Fig. 3b) (utilizing the R package “UpSetR” (version: 1.4.0) (https://CRAN.R-project.org/package=UpSetR) (Conway et al. 2017)). A total of five intersecting genes were obtained and defined as hub genes. These genes were GART, TYMS, PPAT, CTPS1, and PAICS. The expression levels of GART, TYMS, PPAT, and PAICS were significantly higher in DWI-positive samples than in DWI-negative samples, and the expression of CTPS1 was significantly higher in DWI-negative samples than in DWI-positive samples (Fig. 3c).

### Hub Gene and Enrichment Analyses

We analyzed the coexpression network and related functions of the five hub genes using the GeneMANIA database (Fig. 3d), and the network showed 20 related genes for the five hub genes. These genes were found to be functionally associated with nucleoside monophosphate biometabolic processes, ribonucleoside monophosphate metabolic processes, nucleotide biology and nucleobase biosynthesis processes. GSEA enrichment analysis of the hub genes was performed to further explore their signaling pathways. The analysis revealed that CTPS1 was significantly enriched in 4 signaling pathways, GART was significantly enriched in 32 signaling pathways, PAICS was significantly enriched in 29 signaling pathways, PPAT was significantly enriched in 33 signaling pathways, and TYMS was significantly enriched in 22 signaling pathways. The top five most significantly enriched signaling pathways for each gene were visualized using the R package “enrichplot” (version: 1.18.3) (https://yulab-smu.top/biomedical-knowledge-mining-book/) (Zhang et al. 2019) (Fig. 3e–i). CTPS1 was significantly enriched for neuroactive ligand receptor interactions; GART, PAICS, and PPAT were significantly enriched for the MAPK signaling pathway, NOD-like receptor signaling pathway, endocytosis, etc. TYMS was significantly enriched for natural killer cell-mediated cytotoxicity, chemokine signaling pathway, neurotrophic factor signaling pathway, and cytokine-cytokine receptor interactions (see Supplementary Tables 13–17 for details).

### Hub Gene Subcellular Localization, Gene Potential Drug Prediction, and Disease Association Analysis

**Fig. 4:**
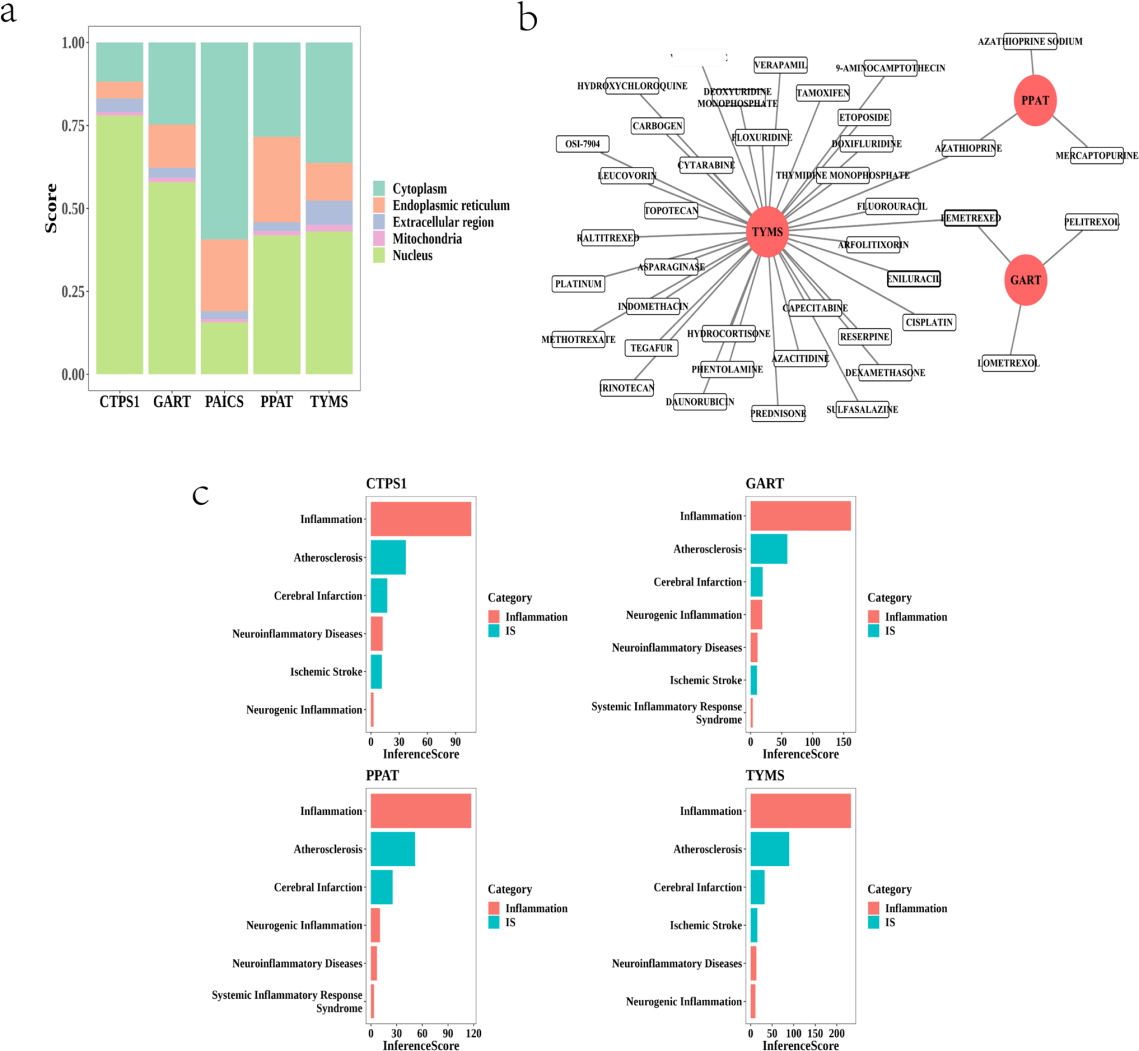
**a** Hub gene subcellular localization prediction scores. Horizontal coordinates are hub genes, and vertical coordinates are subcellular localization prediction scores. **b** Correspondence between hub genes and small molecule drugs. Red circles represent hub genes, and white rectangles represent small molecule drugs. **c** Impact scores corresponding to hub genes and diseases

Five hub genes were analyzed for subcellular localization, and the predicted scores of hub genes in subcellular localization were calculated using the mRNALocater database (http://bio-bigdata.cn/mRNALocater) (Table 4 and Fig. 4a). According to the five highest subcellular localization scores, GART, TYMS, PPAT, and CTPS1 localized to the nucleus, and PAICS localized to the cytoplasm. The interaction of hub genes with potential therapeutic agents was explored for possible new therapeutic targets for the treatment of DWI-negative patients with IS. The results showed that PAICS and CTPS1 had no corresponding small-molecule drugs associated with them, whereas the other pivotal genes predicted multiple small-molecule drugs (Table 5). Thirty-eight small-molecule drugs were predicted to be associated with TYMS, and PPAT and GART were each predicted to have three small-molecule drugs associated with them. In the hub gene and small molecule drug network diagram (Fig. 4b), 44 nodes (3 hub genes, 41 drugs) and 43 edges were included. The analysis revealed that azathioprine was predicted for both TYMS and PPAT. We also used the CTD database (https://ctdbase.org/) to explore the diseases that might be associated with hub genes (GART, TYMS, PPAT, CTPS1, and PAICS). PAICS could not be queried for the corresponding disease according to the CTD database. GART, TYMS, PPAT, and CTPS1 had the strongest association with inflammation, followed by atherosclerosis and cerebral infarction (Fig. 4c and Supplementary Table 18).

### TF-mRNA, miRNA‒mRNA, and ceRNA Network Construction and Expression Validation

**Fig. 5:**
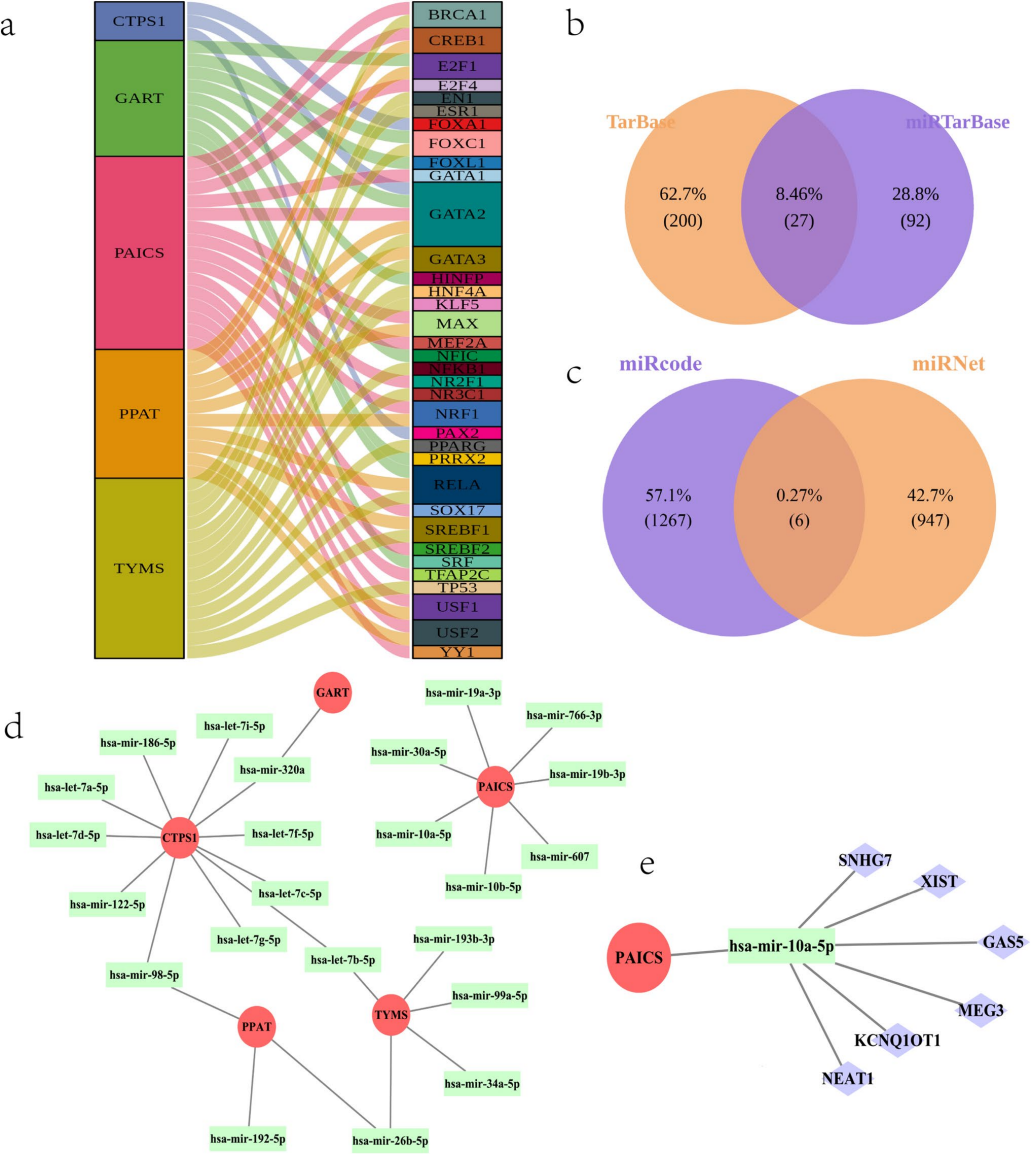
**a** TF-mRNA mulberry diagram. Left side represents a hub gene, and right side represents a TF. **b** miRNA‒mRNA relationship pair intersection. **c** Hub gene and miRNA correspondence. Red circle represents a hub gene, and green rectangle represents a miRNA. **d** lncRNA-miRNA relationship pair intersection. **e** Hub gene and miRNA and lncRNA correspondence. Red circles represent hub genes, green rectangles represent miRNAs, and purple rectangles represent lncRNAs

To investigate the upstream and downstream regulators of hub genes, we performed a Sankey map analysis of the TF-mRNA network using the R packages “ggalluvial” (version: 0.12.5) (Luo et al. 2022) and “ggplot2” (version: 0.9.3.1) (http://had.co.nz/ggplot2) (Ito and Murphy 2013) and predicted 3 TFRNAs for CTPS1, 9 TFRNAs for GART, 15 TFRNAs for PAICS, 10 TFRNAs for PPAT, and 14 TFRNAs for TYMS (Fig. 5a and Table 6). Predicted miRNAs using the TarBase and miRTarBase databases revealed a total of 27 intersecting miRNA‒mRNA pairs (Supplementary Table 19), which contained 5 mRNAs and 23 miRNAs (Fig. 5b). In the hub gene and miRNA regulatory network, 28 nodes (5 mRNAs and 23 miRNAs) and 27 edges were included (Fig. 5c). The black line connects the hub genes represented by red circles to the miRNAs represented by green squares, indicating their interconnections with upstream and downstream relationships (Supplementary Table 20). The above obtained lncRNA‒miRNA relationship pairs were intersected. The analysis revealed that a total of 6 lncRNA‒miRNA pairs intersected (Supplementary Table 21), and this intersection contained 1 miRNA (hsa-mir-10a-5p) and 6 lncRNAs (GAS5, SNHG7, KCNQ1OT1, NEAT1, MEG3, and XIST) (Fig. 5d). The complete lncRNA‒miRNA-mRNA regulatory network contained 6 lncRNA‒miRNA-mRNA relationship pairs (Supplementary Table 22). It contained 8 nodes, 7 edges, 1 miRNA, 1 mRNA, and 6 lncRNAs (Fig. 5e). This implies that all six lncRNAs (SNHG7, XIST, GAS5, MEG3, KCNQ1OT1, and NEAT1) may regulate the imaging profile of DWI after IS through the expression of the miRNA-10a-5p regulatory gene PAICS and that this may be closely related to the immunological and metabolic pathological processes after the onset of IS.

### Expression Validation

**Fig. 6:**
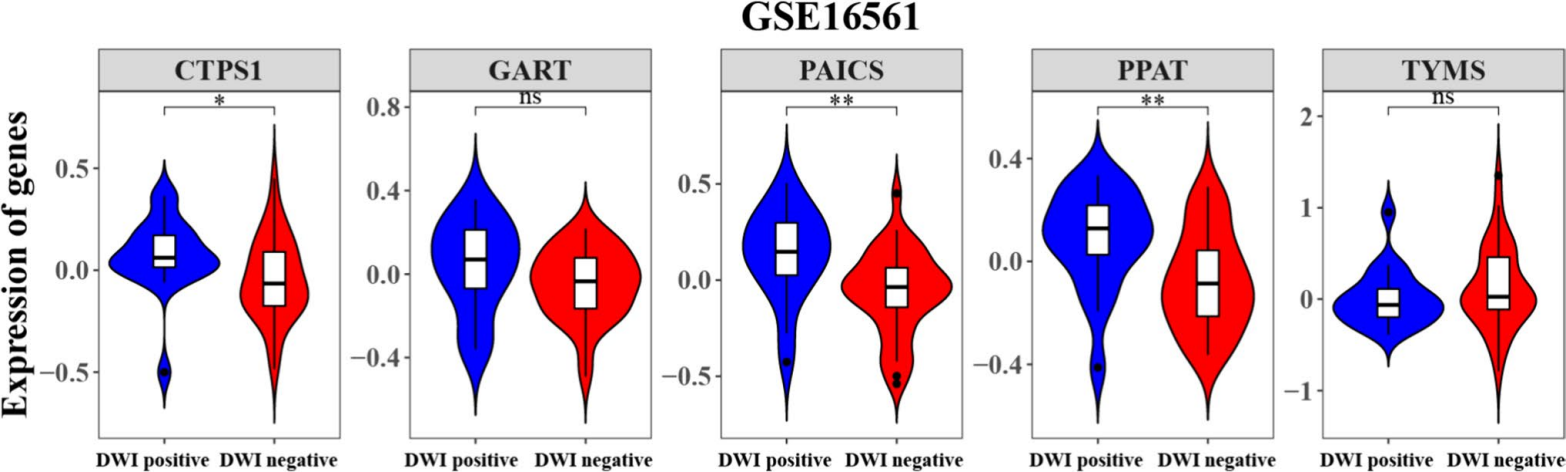
Hub genes in the validation set based on their expression in DWI-negative and DWI-positive patients (****p < 0.0001, ***p < 0.001, **p < 0.01, *p < 0.05, ns stands for *p* > 0.05, where *p* is the *p* value calculated by Wilcoxon test value)

In summary, GART, PAICS, PPAT, and TYMS expression levels were significantly higher in DWI-positive samples than in DWI-negative samples, while CTPS1 expression was higher in DWI-negative samples than in DWI-positive samples. Then, the differences in the five hub genes in the validation set (GSE16561) between DWI-negative and DWI-positive patients were investigated. Based on the box plot of the expression of the genes in the DWI-negative and DWI-positive samples (Fig. 6), PAICS, PPAT, and CTPS1 expression levels were significantly higher in DWI-positive samples than in DWI-negative samples. There were differences in the hub genes in the discovery set and validation set. However, the trend of PAICS and PPAT expression was the same in the discovery set and validation set, and the differences were significant. Ultimately, we think that PAICS and PPAT might be the true hub genes with significant difference.

### Clinical Trial

**Fig. 7:**
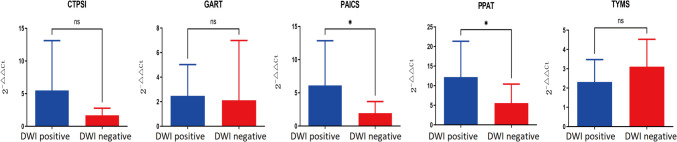
PCR plots of the hub gene in patients with DWI-positive ischemic stroke and in patients with DWI-negative ischemic stroke (*p < 0.05, ns stands for *p* > 0.05)

In order to further verify our conjecture about the hub gene, we collected more whole blood specimens from clinical patients for further verification. We collected whole blood specimens from patients who had symptoms of neurological deficits at the time of admission, were considered to be diagnosed with acute ischemic stroke, and were within 3 days of the onset of the disease, and a total of 30 specimens were collected, of which 15 specimens with positive DWI results and 15 specimens with negative DWI results were collected, and these serum specimens were subjected to reverse transcription-PCR (RT-PCR) for the detection of the expression of the individual hub genes, and data analysis and graphing were performed using the GraphPad Prism 10.0.0 software, and *T*-test was used to statistically analyze the data of the two groups, and *p* < 0.05 was regarded as a statistical difference. All clinical specimens were approved by the Ethics Committee of the Second Affiliated Hospital of Kunming Medical University, and informed consent was signed by the patients or their families. The results are shown in Fig. 7. The raw data are detailed in Supplementary Table 23. We were able to see that the expression of CTPS1, TYMS, PAICS, and PPAT in the blood specimens of clinical patients was consistent with the expression trend of our validation set, but only PAICS and PPAT were significantly differentially expressed, which might be able to indicate that they could be present as hub genes.

## Discussion

In 2010, the American Academy of Neurology (AAN) published an evidence-based guideline on DWI in the diagnosis of AIS, suggesting that DWI should be the most accurate diagnosis of acute IS (Schellinger et al. 2010). DWI examination is an important diagnostic tool for AIS with high sensitivity (88–100%) and high specificity (95–100%) (Simonsen et al. 2015; Sorensen et al. 1996). However, in recent years, many DWI examinations were found to be mismatched with clinical symptoms, mostly related to time of onset, site of onset, and symptoms of onset (Regenhardt et al. 2022). Some studies have started to look at homocysteine levels (Yuan et al. 2019) and plasma osmolality (Aiba et al. 2019) to further diagnose AIS with DWI negative. In the present study, we aimed to identify immunometabolic markers that are different in DWI-negative patients than in DWI-positive patients to further explore their role in IS.

Cytidine nucleotide synthase 1 (CTPS1) is an approximately 67 kDa protein consisting of 591 amino acids located on human chromosome 1p34.1. It is the rate-limiting enzyme in the de novo synthesis of cytosine nucleotides and in the recycling synthesis of uridine (Hatse et al. 1999). It is a CTP synthase that catalyzes the biosynthesis of triphosphate, UTP, and glutamine and plays a key role in DNA synthesis and cell cycle inhibition (Ostrander et al. 1998; van Kuilenburg et al. 2000). Studies have shown that CTPS1 protein expression is barely detectable in the cytosol of immunodeficient patients. Moreover, CTPS1 expression is low in resting T cells and upregulated in activated T cells. Furthermore, CTPS1-deficient T and B cells show poor value addition and proliferation defects due to G1 inhibition. These findings confirm that CTPS1 is required for an effective immune response, a process that occurs primarily through the critical regulation of pyrimidine de novo synthesis (Martin et al. 2014). Our study showed that CTPS1 was significantly upregulated in DWI-negative patients after IS. Additionally, in recent years, it has been found that the direct or indirect immune response induced by the onset of IS plays a key role in its prognosis, with T cells infiltrating extensively around the ischemic semidark zone and gradually spreading to the center of the lesion within 24 h of IS onset (Zhang et al. 2022a, b, c). CD8 + T lymphocytes are involved in neuronal cell injury through the release of cytotoxic proteases (Gu et al. 2015), and CD4 + and CD8 + T cells can increase IS and brain injury after cerebral ischemia and reperfusion by increasing inflammatory responses and thrombosis (Yilmaz et al. 2006). We conjecture that the upregulation of CTPS1 may be involved in the proliferative infiltration process of T cells after IS and affect the prognosis of IS patients by strengthening immunity. However, this needs to be confirmed by further refinement of relevant experiments. CTPSI may also play an important role in in-stent restenosis (ISR). It has been found that the inhibition of CTPS1 expression can selectively block smooth muscle cell proliferation and does not promote endothelial thickening of the vasculature after injury. This could serve as a new target for the treatment of anti-endothelial regeneration and prevention of in-stent restenosis after cardiovascular interventional stenting (Tang et al. 2013).

Phosphoribosylaminoimidazolesuccinocarboxamide synthase (PAICS) is a bifunctional enzyme that catalyzes the de novo synthesis of purines with 4-(N-succinimidoylformamide)-5-aminoimidazole ribonucleotide synthetase activity in its N-terminal region and 5-aminoimidazole ribonucleotide carboxylase activity in its C-terminal region (Chakravarthi et al. 2018). PAICS also interacts with the structural domains of NACHT and WD repeats (Nwd1) to regulate the assembly of purinosomes in the spatiotemporal differentiation of neural stem/progenitor cells (NSPCs). It also induces premature mitotic differentiation of neural stem cells and inhibits neuronal migration (Yamada et al. 2020). After IS onset, NSPCs can undergo proliferation, differentiation, and migration in the subgranular region of the lateral ventricles and hippocampal dentate gyrus to form functional mature neurons (Hatakeyama et al. 2020; Jin et al. 2001), which are closely related to neurological rehabilitation after IS.

A previous study found that the glycine nucleotide transcarbamate (GART) gene is located on human chromosome 21 and is one of the key functional proteins in the three steps of purine de novo synthesis (Moore et al. 1977). Abnormalities in the synthesis of purines lead to inborn metabolic defects in humans and are usually closely associated with mental retardation, sensorineural hearing loss, and optic nerve atrophy. It has been found to be abnormally overexpressed in individuals with Down syndrome and is thought to be involved in the phenotype of Down syndrome (Knox et al. 2009). Phosphoribosyl pyrophosphate amidotransferase (PPAT) is a regulatory transmutase that catalyzes the first step in the purine nucleotide biosynthesis pathway (Liu et al. 2020). Our study showed that the expression of GART and PPAT was significantly higher in the DWI-positive group than in the negative group after IS. It has also been shown that the expression of GART and PPAT genes is upregulated in mice after 2 weeks of sustained intake of high-fructose food. The upregulation of GART and PPAT further accelerates the synthesis of an important intermediate product, hypoxanthine ribonucleotide (IMP), from purines and drives the synthesis of adenine ribonucleotide (AMP) from IMP. IMP then finally undergoes catabolic metabolism to uric acid by increasing the activity of xanthine oxidoreductase (XOR) (Zhang et al. 2022b). Increased uric acid not only accelerates atherosclerosis by increasing overall oxidative stress through increased endothelial nitric oxide synthase (eNOS) and 8-hydroxy-2′-deoxyguanosine (8-OHdG) activity (Huh et al. 2012; Song and Zhao 2018) but also induces endothelial cell senescence and death through local activation of the renin-angiotensin system. This leads to obvious lesions on endothelial surface fibrosis, including the emergence of a large number of foam cells, inflammatory cell aggregation, and lipid deposition, ultimately leading to atherosclerosis and even stenosis (Yu et al. 2010). Atheromatous plaque formation and progression of stenosis can further contribute to the development of IS.

Mitosis-activated protein kinase (MAPK) is involved in the regulation of various biological processes, such as cell proliferation, differentiation, migration, and apoptosis (Imajo et al. 2006). Extracellular-regulated kinases 1 and 2 (ERK1/ERK2), c-Jun-N-terminal kinases (JNKs), and p38 (MAPK14) are the three main MAPK pathways (Slattery et al. 2018). After the onset of IS, ERK2 expression is upregulated, leading to cerebral edema by increasing blood‒brain barrier (BBB) permeability (Schanbacher et al. 2022). It can also lead to increased mRNA expression of intercellular adhesion molecule (ICAM1) and vascular adhesion molecule 1 (VCAM1), which can exacerbate poststroke inflammatory cell infiltration (Jayaraj et al. 2019) and cause further deterioration. JNKs and p38 are involved in postischemic inflammatory factor activation and play important roles in apoptosis, inflammation, and cell cycle regulation (Cuenda and Rousseau 2007; Dhanasekaran and Reddy 2008; Morrison 2012). Nucleotide-binding and oligomerization domain (NOD)-like receptors (NLRs) play essential roles in various diseases. NLR family-containing inflammasomes mediate postischemic inflammatory responses through priming, activation, and interleukin-1β release (Zhang et al. 2022c), further aggravating brain damage.

Immunomodulatory therapies have clear therapeutic implications for a variety of immune-mediated neurological disorders, such as myasthenia gravis (MS), Guillain Barre syndrome (GBS), and CNS demyelinating disorders. Azathioprine is a potent immunosuppressant that is converted to 6-mercaptopurine (its active form) in the liver and kidneys and produces immunosuppression by binding purine analogs to DNA strands interfering with DNA synthesis (Grzechocinska et al. 2023). Thus, it inhibits the proliferation of lymphocytes to prevent the conversion of antigen-sensitive lymphocytes into immune cells (Sahasranaman et al. 2008). After the onset of IS, damage-associated molecular patterns (DAMPs) released from hypoxic death of the affected brain tissue activate the innate immune system, stimulate microglia to activate and polarize into phagocytes, release large amounts of proinflammatory factors, and further exacerbate systemic inflammation (Hu et al. 2012). Studies have shown that immunosuppression in the acute phase of IS can be used as a protective mechanism to counteract the excessive inflammatory response after cerebral hypoxic injury, but prolonged immunosuppression increases the risk of infections in patients (Galati et al. 2021). It may be possible that immunosuppressive therapy can be used as an effective treatment for inhibiting inflammation in AIS, but further investigations are needed regarding the timing of its application and its side effects and other implications.

Although we screened for differentially expressed genes from both immune and metabolic aspects and performed separate gene enrichment analyses, we must acknowledge some limitations of this study. First, we did not find a DWI-negative IS dataset and could only screen from the IS dataset. Second, our specimen size was relatively small and should be validated later using larger sets of data. Additionally, the data were derived from patient blood specimens, the reliability of which needs to be verified in later studies. Third, our screened hub genes have not yet been found in IS studies, and their specific effects and possible mechanisms of action need to be further supported by basic or clinical trials.

## Results

In this study, we identified five immunometabolism-related hub genes in IS, namely, GART, TYMS, PPAT, CTPS1, and PAICS, based on autosequencing data, combined with difference-in-differences and PPI analyses. The five hub genes were functionally related to nucleotide biosynthesis, ribonucleoside monophosphate metabolism, and nucleobase biosynthesis. The GSEA enrichment analysis of the hub genes revealed that GART, PAICS, and PPAT were significantly enriched in the MAPK signaling pathway, NOD-like receptor signaling pathway, and endocytosis. By subcellular localization analysis, GART, TYMS, PPAT, and CTPS1 were found to be localized to the nucleus, and PAICS was localized to the cytoplasm. By drug prediction analysis, GART, TYMS, and PPAT can predict a variety of small molecule drugs, and azathioprine is predicted by TYMS and PPAT. Construction of a ceRNA network of hub genes indicated that hsa-10a-5p acts as a hub miRNA connecting PAICS and multiple lncRNAs in this study. Overall, this study integrates RNA-seq data on the roles of IRGs and MRGs in IS based on bioinformatics tools to explore the biological pathways of these hub genes and to develop new therapeutic strategies for IS.

## Supplementary Information

Below is the link to the electronic supplementary material.

Supplementary file1 (JPG 1986 KB)

Supplementary file2 (XLS 21590 KB)

Supplementary file3 (XLS 68 KB)

Supplementary file4 (XLS 63 KB)

Supplementary file5 (XLS 5877 KB)

Supplementary file6 (XLS 2710 KB)

Supplementary file7 (XLS 622 KB)

Supplementary file8 (XLS 19 KB)

Supplementary file9 (XLS 2711 KB)

Supplementary file10 (XLS 658 KB)

Supplementary file11 (XLSX 46 KB)

Supplementary file12 (XLSX 10 KB)

Supplementary file13 (XLSX 18 KB)

Supplementary file14 (XLS 2 KB)

Supplementary file15 (XLS 13 KB)

Supplementary file16 (XLS 12 KB)

Supplementary file17 (XLS 14 KB)

Supplementary file18 (XLS 8 KB)

Supplementary file19 (XLSX 2010 KB)

Supplementary file20 (XLSX 7 KB)

Supplementary file21 (XLS 20 KB)

Supplementary file22 (XLSX 6 KB)

Supplementary file23 (XLSX 6 KB)

Supplementary file24 (XLS 23 KB)

Supplementary file25 (PDF 386 KB)

Supplementary file26 (PDF 164 KB)

## Data Availability

All data analyzed during this study are included in this published article.
