# Supplementary figures and images for: Identification of Potential Biomarkers for Patients with DWI-Negative Ischemic Stroke
Source: J Mol Neurosci. 2024 Jul 12;74(3):68. doi: 10.1007/s12031-024-02229-z (PMC11245437; doi:10.1007/s12031-024-02229-z)

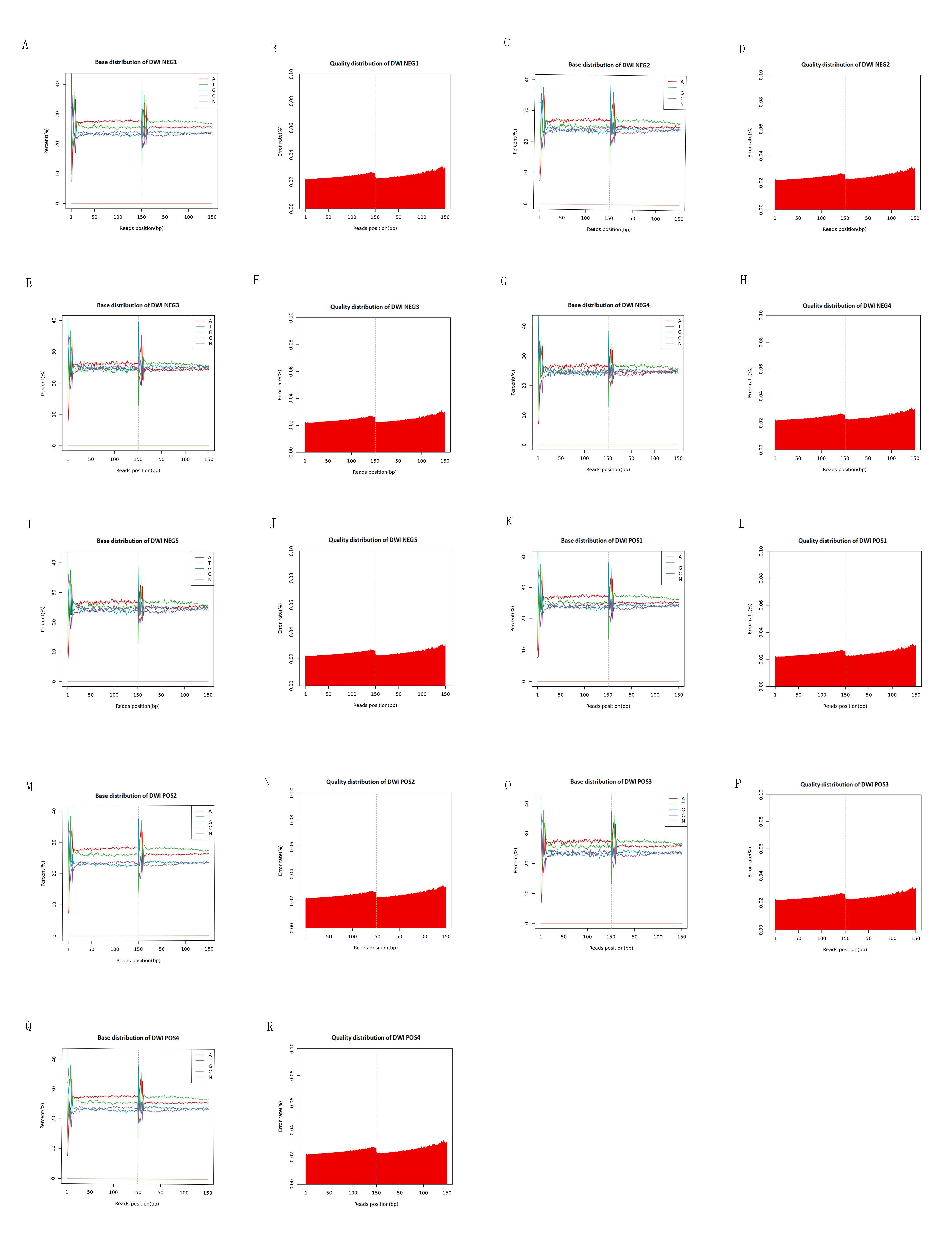

Supplement: Supplementary file 1 — Supplementary file1 (JPG 1986 KB) [file 12031_2024_2229_MOESM1_ESM.jpg]
