# Supplementary material for: Identification of Potential Biomarkers for Patients with DWI-Negative Ischemic Stroke
Source: J Mol Neurosci. 2024 Jul 12;74(3):68. doi: 10.1007/s12031-024-02229-z (PMC11245437; doi:10.1007/s12031-024-02229-z)
Supplement: Supplementary file 25 — Supplementary file25 (PDF 386 KB) [file 12031_2024_2229_MOESM25_ESM.pdf]

医学伦理委员会审查批件

审-PJ-科-2022-124

项目名称	TLR4 介导的脑缺血再灌注损伤炎症反应中 lncRNAs 及相关 ceRNA 作用机制研究		
项目来源	昆明医科大学第二附属医院		
申请科室	神经内科	主要研究者	王颖
研究类型	☐药物临床试验　☐器官移植　☐人类辅助生殖　☑科研课题<br>☐医疗器械、设备　☐医疗技术　☐其他		
审查类别	初始审查	审查方式	☑会议审查　☐快速审查
审查日期	2022.04.26	审查地点	医院学术交流中心六号会议室
审查委员	见会议签到表		
审查文件	1.主要研究者专业履历<br>2.研究经济利益声明<br>3.国家自然科学基金委员会资助项目计划书<br>4.临床研究工作方案（1.0 版/2022 年 3 月 15 日）<br>5.知情同意书（1.0 版/2022 年 3 月 15 日）		
年度/定期跟踪审查频率	12 个月	有效期	截止日期：　2023.04.25

审查意见：

经伦理委员会讨论决定：批准在本中心开展研究。

主任/副主任委员签字：

昆明医科大学第二附属医院医学伦理委员会（盖章）

2022 年 5 月 5 日

注意事项：

1.如试验预期未完成，请提前一个月提交《研究进展报告》申请延长有效期；
2.自同意研究之日起，请按照跟踪审查频率要求，提前一个月提交《研究进展报告》；
3.发生严重不良事件，请及时提交《严重不良事件报告表》；
4.研究过程中变更主要研究者，或者对临床研究方案、知情同意书等进行修改，请提交《修正案审查申请》；
5.研究纳入了不符合纳入标准或符合排除标准的受试者，符合中止试验规定而未让受试者退出研究，给予错误治疗或剂量，给予方案禁止的合并用药等没有遵从方案开展研究的情况，请提交《违背方案报告》；
6.暂停或提前终止临床研究，请及时提交《暂停/终止研究报告》；
7.完成临床研究，请提交《研究完成报告》。

声明：本伦理委员会的职责、人员组成、运行和记录遵循国家药监局和国家卫生健康委颁布的药物临床试验质量管理规范（GCP）和 ICH-GCP 伦理审查原则，并遵守中国的相关法律及法规。

扫描全能王
3亿人都在用的扫描App
