# Supplementary material for: Identification of Potential Biomarkers for Patients with DWI-Negative Ischemic Stroke
Source: J Mol Neurosci. 2024 Jul 12;74(3):68. doi: 10.1007/s12031-024-02229-z (PMC11245437; doi:10.1007/s12031-024-02229-z)
Supplement: Supplementary file 26 — Supplementary file26 (PDF 164 KB) [file 12031_2024_2229_MOESM26_ESM.pdf]

伦理审查意见

审-YJ 2024-17

项目名称	TLR4 介导的脑缺血再灌注损伤炎症反应中 lncRNAs 及相关 ceRNA 作用机制研究		
项目申办方	国家自然科学基金		
主要研究者	王颖	申请研究科室	神经内科
审查类别	年度/定期跟踪审查	审查方式	快速审查
审查日期	2024 年 02 月 01 日	审查地点	不适用
审查委员	杨晋辉、周泽平		
审查文件	研究进展报告		

审查意见

根据《涉及人的生物医学研究伦理审查办法》、《药物临床试验质量管理规范》、《医疗器械临床试验规定》、WMA《赫尔辛基宣言》和 CIOMS《人体生物医学研究国际道德指南》的伦理原则，经本伦理委员会审查，意见如下：

同意继续开展临床试验。有效期至 2025 年 04 月 25 日，如试验预期未完成，请提前一个月提交《研究进展报告》申请延长有效期。

按审查意见修改后的文件，或对审查意见不同观点的陈诉，请提交“复审申请”，方案/知情同意书请注明新的版本号和版本日期，并以阴影和/或下划线方式标注修改部分，报伦理委员会审查，经批准后执行。

调整的年度/定期跟踪审查频率	12 个月
伦理委员会<br>（盖章）	昆明医科大学第二附属医院医学伦理委员会
主任委员或副主任委员签字<br>（盖章）	[signature]
日期	2024.2.1
